# Is Lipid Accumulation Product Associated with an Atherogenic Lipoprotein Profile in Brazilian Subjects?

**DOI:** 10.5935/abc.20180054

**Published:** 2018-04

**Authors:** Flavia De Conti Cartolano, Caroline Pappiani, Maria Camila Prupper de Freitas, Antonio M. Figueiredo Neto, Antônio Augusto Ferreira Carioca, Nágila Raquel Teixeira Damasceno

**Affiliations:** 1 Faculdade de Saúde Publica da Universidade de São Paulo - São Paulo, SP - Brazil; 2 Instituto de Física da Universidade de São Paulo - São Paulo, SP - Brazil

**Keywords:** Cardiovascular Diseases, Lipoproteins, HDL, Lipoproteins, LDL, Insulin Resistance, Dyslipidemias, Adults, Risk Factors

## Abstract

**Background:**

Lipid accumulation product (LAP), a simple and low-cost tool, is a novel biomarker of central lipid accumulation and represents a potential surrogate marker for atherogenic lipoprotein profile. However, its association with lipoprotein subfractions has not been described in the literature.

**Objective:**

To determine whether LAP index could be used as a marker of low- and high-density lipoprotein (LDL and HDL) size in Brazilian individuals.

**Methods:**

This cross-sectional study included patients (n = 351) of both sexes and age between 30-74 years. Clinical and sociodemographic data and family history of diseases were evaluated. Lipoprotein size, and levels of total cholesterol (TC), lipoproteins, apolipoprotein AI and B (APO AI/APO B), glucose, insulin, insulin resistance index (HOMA-IR) and non-esterified fatty acids (NEFA) were assessed in blood samples. LAP was calculated by the formulas [(waist circumference_[cm]_-58) × (triglycerides_[mmol/L]_) for women and (waist circumference _[cm]_-65) × (triglycerides _[mmol/L]_) for men]. The association between LAP and metabolic parameters were tested by linear trend (general linear model, GLM test) before and after multiple adjustments for potential confounders (sex, age, smoking, statin, fibrate, and hypoglycemic drugs) at significant level p < 0.05.

**Results:**

LAP was positively associated with TC, APO B, NEFA, glucose, insulin and HOMA-IR values, and negatively associated with HDL-C. Higher central lipid accumulation was corelated with higher percentage of intermediate HDL and of small LDL and HDL and less amount of large HDL. LDL size was also reduced in greater LAP index values. The negative impact of LAP was maintained after adjustment for multiple variables.

**Conclusion:**

LAP was robustly associated with atherogenic profile of lipoprotein subfractions, independently of multiple confounders.

## Introduction

Cardiovascular disease (CVD) is the leading cause of premature morbidity and mortality worldwide, compromising significant private and government resources.^1^ Public policy programs are focused on prevention and modification in traditional risk factors (hypertension, dyslipidemia, smoking, and diabetes *mellitus*), which are the basis of all models of cardiovascular risk prediction. Nevertheless, identification of new risk factors and/or markers for CVD is important to better understand some clinical events that cannot be explained by classical risk factors.

These new biomarkers involve measurable biochemical parameters in serum or plasma, however, cholesterol associated with high-density lipoprotein (HDL-C) and low-density lipoprotein (LDL-C) remain the main lipoproteins monitored to estimate cardiovascular risk in adults.^2^ Currently, biomarkers associated with functionality and structure of lipoproteins - such as their size (small, intermediate, large and phenotypes A and B) - antioxidants (tocopherols, carotenoids), apolipoproteins (Apo B, AI, CII, J, F) and enzymes (Lp-PLA_2_, ACAT) have been investigated.^3-5^ Particularly, small dense LDL have been extensively described by its proatherogenic properties. This particle migrates to the subendothelial space more easily, recruits and activates macrophages, causing their transformation into foam cells and generating fatty streak, a hallmark of atherosclerosis.^4^ Contrary to the well-established atherogenic mechanisms of LDL, functional role of HDL size remains controversial. Small HDL species are described as more antioxidant, anti-inflammatory and more capable to promote cellular cholesterol efflux.^6^ In opposite, Woudberg et al. showed that obesity was associated with reduced large HDL subclasses.^7^ Many of these biomarkers are expensive, require methods technically sophisticated and show limited use in primary health care and prevention of diseases.

Lipid Accumulation Product (LAP) was proposed as a simple, inexpensive and accurate surrogate index to estimate cardiovascular risk^8^ and all-cause mortality.^9^ This index combines anthropometric (waist circumference, WC) and biochemical (fasting triglycerides, TG) parameters, connecting anatomical to physiological changes associated with increased central accumulation of lipids in adults. Kahn^10^ observed in the Third National Health and Nutrition Examination Survey (NHANES III) that LAP index evidenced the negative effect of large WC possibly related with small dense LDL, although direct measurement of LDL size has not been done. The validity and superiority of LAP to identify cardiovascular risk, metabolic syndrome, diabetes *mellitus* and insulin resistance have been compared with body mass index (BMI), WC and waist-to-hip ratio.^9-13^ Despite the negative impact of LAP on glucose metabolism, monitored principally in postmenopausal^13,14^ and polycystic ovary syndrome women,^15,16^ its association with the size of lipoproteins has not been directly evaluated and reported yet.

Previous studies based in LAP confirmed its association with classical risk factors for CVD.^17-20^ Therefore, the aim of this study was to extend current knowledge of LAP, by evaluating the impact of this parameter on LDL and HDL size, considering the potential influence of confounders.

## Methods

### Subjects

Three hundred fifty-one adults of both sexes and multiple cardiovascular risk factors were selected for this cross-sectional study after complete clinical evaluation and electrocardiogram (ECG). These subjects were recruited from the Research Center located at the University Hospital of the University of Sao Paulo. The non-probabilistic sampling was employed. According to inclusion criteria, the subjects included in the study were 30-74 years old and had at least one of the risk factors for CVD - dyslipidemia, diabetes *mellitus*, and/or hypertension. Pregnant or lactating women, individuals who participated in other studies, had severe hepatic or renal disease, type 1 diabetes mellitus, illicit drug users, alcoholics, and individuals under lipid-lowering drugs introduced or changed 30 days before blood collection were not enrolled in this protocol. This study was approved by the Research Ethics Committee of the University Hospital (n 1126/11) and the School of Public Health, University of Sao Paulo (n 2264) and all procedures followed the standards of the Declaration of Helsinki of 1975, revised in 2008. All subjects gave their written informed consent.

### Demographic and clinical profile

Trained interviewers evaluated the demographic features of participants by a pre-structured questionnaire addressing sex, age, and ethnicity. The clinical evaluation consisted of current information on medical history, family history of chronic diseases (father and mother), and regular use of medication. Smoking was considered when the habit was reported by the subjects, regardless of the amount of cigarettes. Hypertension was confirmed by clinical history, use of antihypertensive medication and systolic (SBP) and diastolic (DBP) blood pressure monitored after at least five minutes at rest and mean of three measures was used for data analysis. Hypertension was defined as SBP ≥ 140 mmHg and/or DBP ≥ 90 mmHg. Type 2 diabetes mellitus was defined by previous diagnosis of diabetes, use of oral hypoglycemic agents and plasma glucose levels higher 100 mg/dl. The Framingham Risk Score (FRS) was calculated as previously described.^21,22^

### Anthropometric parameters

Weight (Kg) and height (cm) were measured to the nearest 0.1 kg and 0.1 cm, respectively, with standard methods and equipment. BMI was calculated as weight (Kg) divided by the square of the standing height (m^2^). The WC was measured using flexible inelastic tape with an accuracy of 1.0-mm (TBW^®^; Sao Paulo, SP, Brazil) without tightening it against the body. Body composition was assessed by bioelectrical impedance (BIA) (Analyzer®, model Quantum II; RJL Systems; Michigan, USA). Body fat percentage was calculated using the Cyprus (Body Composition Analysis System, v. 2.5; RJL Systems®; Detroit, MI, USA) program, which considered sex, age, weight, height, level of physical activity, resistance and reactance. All measurements were performed in duplicate by trained staff.

### Blood samples

After fasting (12 h), blood samples (20 mL) were collected. For analyses using plasma, blood was collected in vacutainer tubes containing ethylenediaminetetraacetic acid (EDTA; 1.0 µg/mL). The protease inhibitors aprotinin (10.0 µg/ml), benzamidine (10.0 µM), phenylmethylsulfonyl fluoride (PMSF; 5.0 µM) and the antioxidant butylated hydroxytoluene (BHT; 100.0 µM) were added to the samples. Plasma and serum were separated by centrifugation (3,000 rpm; 10 min; 4ºC) and samples were kept frozen (−80 ºC) until analysis.

### Biochemical Analysis

Plasma TG, total cholesterol (TC), and HDL-C levels were measured using commercial kits (Labtest; Lagoa Santa, MG, Brazil). LDL-C levels were calculated using the Friedewald equation for subjects who had TG lower than 400 mg/dl.^23^ Apolipoproteins B and AI (Apo B and Apo AI) were determined using standard methods (APO A1 and APO B Autokits, Randox; Kearneysville, WV, USA). Non-esterified fatty acids (NEFA) levels were determined using the Free Fatty Acid Quantification kit (Wako Chemicals - USA Inc.; Richmond, VA, USA). Glucose levels were determined using an enzymatic and colorimetric kit (Glucose PAP Liquiform; Labtest; Lagoa Santa, MG, Brazil). Plasma insulin was detected using the commercial Human Insulin Direct ELISA kit (Life Technologies; Grand Island, NY, USA). Insulin resistance was calculated using the homeostatic model assessment-insulin resistance (HOMA-IR) formula as follows: HOMA-IR = fasting insulin concentration (U/mL) x fasting glucose (mmol/L)/22.5.^24^ These parameters were analyzed in duplicate in automatic Cobas system (Hitachi High Technology, Minato-ku, Tokyo, Japan).

The distribution of HDL and LDL subfractions was determined using the Lipoprint supplier system based on nondenaturing polyacrylamide gel. The LDL1 and LDL2 sub-fractions were classified as large LDL, and sub-fractions from LDL3 to LDL7 were classified as smaller and denser particles. The LDL size (nm) was determined and from that, phenotype A (> 25.6 nm, large and less dense LDL) and non-A (≤ 25.6 nm, small dense LDL) pattern were calculated. For HDL particle size, ten sub-fractions were identified, which were classified as large (HDL1 to HDL3), intermediate (HDL4 to HDL7), and small (HDL8 to HDL10) particles.

All analyses were conducted in duplicate and intra- (1-5.8%) and inter- (0.5-15%) assay coefficients of variance were calculated.

### Lipid Accumulation Product (LAP)

LAP was calculated using different formulae for women (WC _[cm]_-58) × (TG _[mmol/L]_) and men (WC _[cm]_-65) × (TG _[mmol/L]_), which include the minimum sex-specific WC values.^8^

### Statistical analysis

Statistical analysis was performed using the Statistical Package for the Social Sciences (SPSS®; v. 20.0) software package. Two-sided P values < 0.05 were considered statistically significant. The Kolmogorov-Smirnov test (p > 0.05) was applied to assess normality of data. Normally distributed continuous variables are presented as mean values and standard deviations (SD), whereas non-normally distributed data are presented as median and 25th and 75th percentiles. Categorical variables are presented as absolute values (n) and percentages (%). Groups were compared using the unpaired Student’s t-test for normally distributed data. Non-normally distributed data were analyzed using non-parametric Mann-Whitney U tests. Categorical variables were compared using the Pearson chi-square or Fisher’s exact test. Subjects were divided into tertiles (T) of the LAP index: T1 ≤ 45.5; 45.5 < T2 ≤ 80.3; and T3 > 80.3. Association between tertiles of LAP index and atherogenic lipoprotein profile were tested in a linear trend test by raw and adjusted models: age and sex (Model A) and age, sex, smoking, use of statin, fibrate, and/or hypoglycemic drugs (Model B). In addition, comparison between groups was performed by analysis of variance (ANOVA or Kruskal-Wallis - with multiple comparisons by Tukey test) after all adjustments (Model B) with significance level at p < 0.05.

## Results

The demographic and clinical characteristics of the 351 subjects grouped by sex are shown in Table 1. The mean age of the subjects was 49.4 years for men (range: 30-72 years) and 54.4 years for women (range: 30-74 years, p < 0.001). Women were older and reported greater use of drugs than men (83.6 *versus* 69.8, respectively, p = 0.001), whereas higher percentage of men were smokers (p = 0.026). More than 80% of the subjects had a prior disease at the time of screening. Hypertension was the most prevalence disease in both genders (56.9% in men and 57.1% in women), which was corroborated by the high percentage of antihypertensive drug users. This profile is in concordance with elevated frequency of hypertension in father, mother or both parents of individuals (62.9% in men and 66.2% in women).

**Table 1:** Demographic and clinical characteristics of subjects by gender

Variables	Total (n = 351)		Men (n = 132)		Women (n = 219)		p
	n	%	n	%	n	%	
Age (years) **	52.5	(10.4)	49.4	(11.1)	54.4	(9.6)	< 0.001
***Smoking*** No	282	80.3	98	74.2	184	84.0	0.026
Current illnesses	306	87.2	114	86.4	192	87.7	0.723
Diabetes mellitus	71	20.2	32	24.2	39	17.8	0.146
Hypertension	200	57.0	75	56.8	125	57.1	0.962
Dyslipidemia	192	54.7	72	54.5	120	54.8	0.964
Drugs	274	78.1	91	69.8	183	83.6	0.001
Statin	98	27.9	28	21.2	70	32.0	0.030
Antihypertensive	181	51.6	64	48.5	117	53.2	0.370
Hypoglycemic agents	73	20.8	29	22.0	44	20.1	0.674
Fibrate ^§^	9	2.6	3	2.3	6	2.7	0.543
Family history of diseases	320	91.2	122	92.4	198	90.4	0.520
Obesity	64	18.2	28	21.2	36	16.4	0.262
Hypertension	228	65.0	83	62.9	145	66.2	0.526
Acute myocardial infarction	100	28.5	38	28.8	62	28.3	0.924
Stroke	67	19.1	25	18.9	42	19.2	0.956
Diabetes mellitus	134	38.2	49	37.1	85	38.8	0.752
Peripheral vascular disease	25	71	8	6.1	17	7.8	0.548

Comparative analysis of categorical variables was performed by Pearson chi-square or Fisher's exact test

(§) (p < 0.05).

** Data presented as mean and standard deviation. Comparative analysis of continuous variables was performed using the unpaired Student's t-test (p < 0.05)

Table 2 shows results of cardiovascular risk, assessed by FRS, and biochemical and anthropometric variables stratified by sex. The FRS was similar between men (13.6 points) and women (13.5 points), indicating a moderate cardiovascular risk in both groups. Men showed higher values of WC and TG, impacting directly on elevated values of LAP in comparison with women. In contrast, women had higher values of Apo AI, HDL-C and NEFAs. Both groups showed similar profile of BMI and glucose homeostasis evaluated by glucose, insulin and HOMA-IR parameters. The influence of gender on lipid metabolism was confirmed by elevated percentage of small HDL and LDL and reduced percentage of large HDL observed in men. This profile was reinforced by the increase of LDL size in men (26.9 in men *versus* 27.0 in women; p = 0.001) and phenotype A in women (52.3% in men *versus* 70.8% in women; p = 0.001).

**Table 2:** Framingham risk score, biochemical and anthropometric characteristics of subjects by gender

Variables	Total (n = 351)	Men (n = 132)	Women (n=219)	p
FRS (points)	13.5 (4.8)	13.6 (5.0)	13.5 (4.5)	0.941
HDL-C (mg/dl)	37.0 (10.0)	32.0 (7.0)	40.0 (10.0)	< 0.001
LDL-C (mg/dl)	139.0 (38.0)	133.0 (22.0)	41.0 (40.0)	0.092
TG (mg/dl)*	128.0 (94.0 - 188.0)	145.0 (10.06 - 213.0)	121.0 (90.0 - 172.0)	0.001
Apo AI (mg/dl)	132.0 (25.0)	123.0 (33.0)	137.0 (26.0)	< 0.001
Apo B (mg/dl)	104.0 (25.0)	103.0 (23.0)	105.0 (26.0)	0.400
NEFA (mEq/dl)	0.6 (0.3)	0.6 (0.3)	0.7 (0.3)	0.016
Small LDL (%)*	1.6 (0.8 - 4.5)	2.1 (1.0 - 6.3)	1.4 (0.6 - 3.6)	0.003
Large LDL (%)	26.3 (5.4)	26.6 (4.9)	26.1 (5.6)	0.491
Small HDL (%)	19.8 (7.1)	21.1 (6.5)	19.1 (7.4)	0.022
Inter HDL (%)	50.3 (5.1)	51.1 (4.5)	49.8 (5.3)	0.039
Large HDL (%)	29.9 (8.6)	27.8 (7.8)	31.0 (8.8)	0.002
LDL size* (nm)	27.0 (26.5 - 27.2)	26.9 (26.4 - 27.1)	27.0 (26.7 - 27.2)	0.001
Phenotype A (%) **	63.8	52.3	70.8	0.001
Glucose (mg/dl)*	97 (91.0 - 108.0)	98 (91.0 - 113.0)	97 (91.0 - 105.0)	0.358
Insulin (µIU/ml)*	16.3 (12.6 - 22.1)	15.6 (12.7 - 22.5)	16.7 (12.4 - 22.0)	0.791
HOMA-IR *	4.0 (2.9 -5.9)	4.2 (3.1 - 5.9)	4.0 (2.9 - 5.8)	0.596
Weight (kg)	77.9 (68.8 - 93.9)	89.7 (75.8 - 101.7)	72.9 (64.1 - 86.5)	<0.001
WC (cm)	100.5 (13.5)	104.2 (12.7)	98.4 (13.5)	<0.001
Body fat (%)	37.8 (25.2 - 46.0)	23.4 (20.7 - 26.9)	43.4 (38.4 - 49.2)	<0.001
BMI (kg/m^2^)	30.8 (5.9)	30.6 (5.4)	30.9 (6.2)	0.628
LAP *	57.7 (35.4 - 87.2)	68.4 (40.5 - 105.0)	53.2 (35.2 - 81.6)	0.026

Data presented as mean (SD) and median (p25-p75). Comparative analysis was performed by the unpaired Student's t test or Mann-Whitney test

(*) and Pearson chi-square

(**) (p < 0.05). FRS: Framingham Risk Score; TC: total cholesterol; HDL-C: high-density lipoprotein cholesterol; LDL-C: low-density lipoprotein cholesterol; TG: triacylglycerol; Apo AI: apolipoprotein AI; Apo B: apolipoprotein B; NEFA: non-esterified fatty acids; BMI: body mass index; LAP: lipid accumulation product; WC: waist circumference.

Raw and adjusted associations between LAP and other parameters were tested by tertiles (Table 3). LAP was positively associated with TC, Apo B, NEFA, glucose, insulin, and HOMA-IR and, consequently, this association increased with FRS points. Surprisingly, LAP was not corelated with LDL-C. After multiple adjustments for potential confounders (A and B models), the associations between LAP and biochemical parameters were maintained, except for Apo AI.

**Table 3:** Linear trend analysis of Framingham risk score and biochemical variables in lipid accumulation product tertiles

	LAP			Raw data	Model A	Model B
	T1 ≤ 45.5 (n = 117)	45.5 < T2 ≤ 80.3 (n = 117)	T3 > 80.3 (n = 117)	p	p	p
FRS	12.3	13.6	14.6^*§^	< 0.001	< 0.001	< 0.001
TC (mg/dl)	198.2	201.0	216.0^*§^	0.001	< 0.001	< 0.001
HDL-C (mg/dl)	40.7	37.6	32.4^*§^	< 0.001	< 0.001	< 0.001
LDL-C (mg/dl)	139.6	136.1	136.2	0.514	0.660	0.770
Apo AI (mg/dl)	135.6	134.2	127.2	0.012	0.062	0.073
Apo B (mg/dl)	97.5	103.8^*^	111.9^*§^	< 0.001	< 0.001	< 0.001
NEFA (mEq/dl)	0.6	0.6	0.7^*^	0.012	0.002	0.006
Glucose (mg/dl)	96.4	101.8	122.1^*§^	< 0.001	< 0.001	< 0.001
Insulin (µIU/ml)	15.1	19.0^*^	21.0^*^	< 0.001	< 0.001	< 0.001
HOMA-IR	3.6	4.7	6.2^*§^	< 0.001	< 0.001	< 0.001

Model A: adjusted by sex and age. Model B: adjusted by sex, age, smoking, statin, fibrate, and hypoglycemic drugs. FRS: Framingham Risk Score; TC: total cholesterol; HDL-C: high-density lipoprotein cholesterol; LDL-C: low-density lipoprotein cholesterol; Apo AI: apolipoprotein AI: Apo B: apolipoprotein B; NEFA: non‑esterified fatty acids; LAP: lipid accumulation product. Comparison between groups was performed by ANOVA or Kruskal-Wallis and multiple comparisons by Tukey test.

* versus T1,

§ versus T2. Significance level adopted for all analysis p < 0.05.

**Figure 1:**
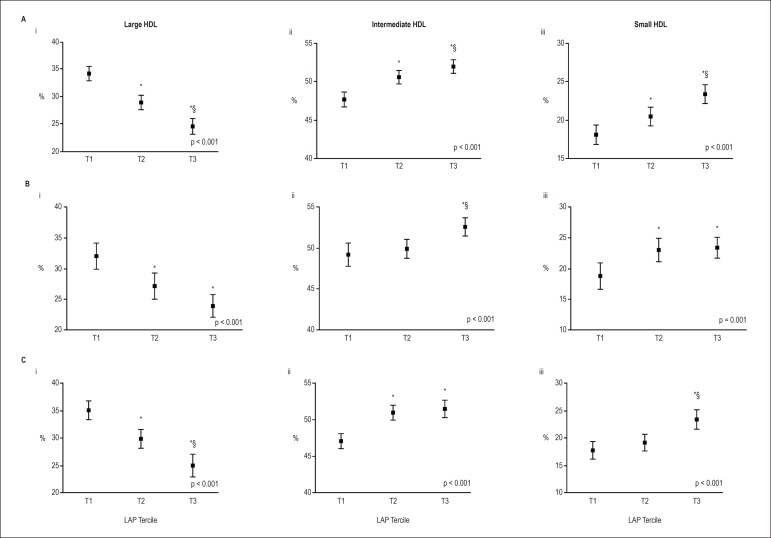
Percentages of large, intermediate, and small HDL (high density lipoprotein) particles, according to the LAP (lipid accumulation protein) tertiles. A) Adjusted by sex, age, smoking, statin, fibrate, and hypoglycemic drugs. B) Men, adjusted by age, smoking, statin, fibrate, and hypoglycemic drugs (n = 132). C) Women, adjusted by age, smoking, statin, fibrate, and hypoglycemic drugs (n = 219). i: Larger HDL. ii: Intermediate HDL. iii: Small HDL. Data are presented as mean and 95% confidence interval. Comparative analysis was performed using the linear trend test. LAP tertiles: T1 ≤ 45.5; 45.5 < T2 ≤ 80.3; T3 > 80.3. HDL - high-density lipoprotein, LAP: lipid accumulation product, % - percentage. Comparison between groups was performed by ANOVA or Kruskal-Wallis and multiple comparisons by Tukey test. *versus T1, *§*versus T2. Significance level adopted for all analysis p < 0.05.

**Figure 2:**
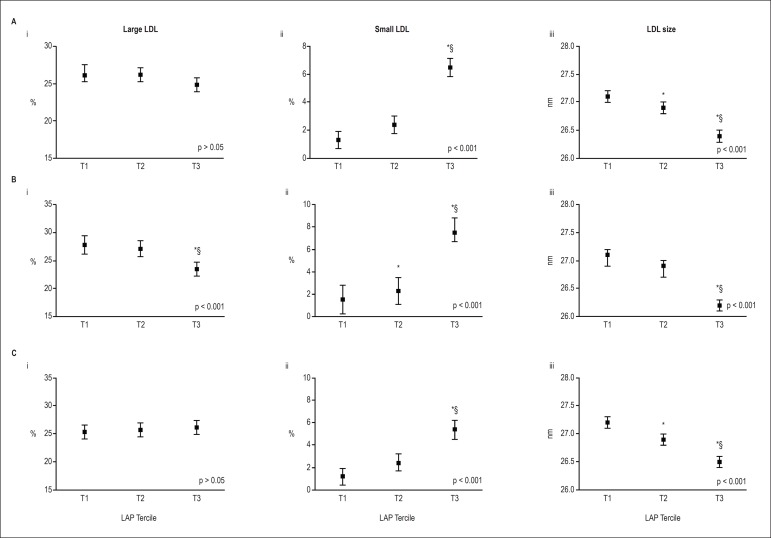
Percentages of large and small LDL particles and LDL size, according to the LAP tertiles. A) Adjusted by sex, age, smoking, statin, fibrate, and hypoglycemic drugs. B) Men, adjusted by age, smoking, statin, fibrate, and hypoglycemic drugs (n = 132). C) Women, adjusted by age, smoking, statin, fibrate, and hypoglycemic drugs (n = 219). i: Large LDL. ii: Small LDL. iii: LDL size. Data are presented as mean and 95% confidence interval. Comparative analysis was performed using the linear trend test. LAP tertiles: T1 ≤ 45.5; 45.5 < T2 ≤ 80.3; T3 > 80.3. HDL: high-density lipoprotein; LAP: lipid accumulation product; %: percentage Comparison between groups was performed by ANOVA or Kruskal-Wallis and multiple comparisons by Tukey test. *versus T1, *§*versus T2. Significance level adopted for all analysis p < 0.05.

Also, central lipid accumulation was positively associated with the percentage of intermediate and small HDL subfractions in both total (Figure 1A) and sex-stratified sample (Figures 1B, 1C) after adjustment for age, smoking, and use of statin, fibrate and hypoglycemic drugs. Similar results were found for small LDL, i.e., individuals in lowest, in the middle and in the highest tertile showed about 1.5%, 2.3% and 7.5% of small LDL, respectively (p < 0.001) (Figure 2Aii). Higher differences were seen in men (Figure 1Bi).

LDL size and percentage of large HDL were both negatively associated with LAP. In total sample, this difference was nearly 10 points for large HDL - 34.2% in T1 and 24.5% in T3 (Figures 1Ai, Bi, Ci). Associations between LAP index and large LDL were found in men (Figure 2Bi), but not in total sample nor in women, demonstrating a sex-dependent relationship for this subfraction.

## Discussion

Based on this cross-sectional study, LAP has a significant association with classical and new cardiovascular biomarkers. These associations were especially important when LAP index was corelated to size of the LDL and HDL particles.

Previously, Kahn and Valdez^8^ evaluated a cross-sectional sample from the NHANES III and reported that individuals with high WC and TG levels were more likely to show inadequate levels of HDL-C, Apo B, fasting insulin, and glucose. Later, Kahn^11^ confirmed that the LAP was superior to BMI in indicating adults with diabetes mellitus and for predicting imbalance in glucometabolic variables (HOMA-IR, fasting glucose, and glycated hemoglobin). Similar results were found in studies conducted in other countries, in which LAP was a better marker of glucose imbalance and a stronger predictor of DM than BMI.^13-20^ The present study confirms that LAP is sensitive to identify dysfunctions related to glucose metabolism, even after adjustment for drug use and multiple confounders.

The relevance of LDL-C in the development of atherosclerosis has been recognized. However, some individuals with normal LDL-C levels have cardiovascular events, indicating that other risk factors related or not with LDL exert a role in the atherosclerotic process. Epidemiological evidence shows that an increased proportion of small and dense LDL particles is strongly associated with the risk of coronary heart disease.^25^ Individuals with elevated plasma concentrations of small and dense LDL are at 3-7 times greater risk to develop coronary artery disease (CAD), independent of the LDL-C level.^5^ Smaller and denser LDL, known as phenotype B, has been proposed as a more atherogenic sub-fraction than large LDL. Smaller particles remain for a longer time in plasma and shows reduced affinity for the B/E receptor.^25^ Phenotype-B LDL is highly recognized by scavenger receptor, and therefore is more susceptible to migration to the subendothelial layer and oxidation.^4,5^ Despite that, the relationship between LAP and LDL size has not been described in the literature. Our results showed that small LDL particles and LDL size were positively and negatively associated with LAP, respectively, even if LDL-C was not related to LAP. Mirmiran et al.^26^ also didn’t find any correlation between LAP and LDL-C.

Reinforcing the negative role of small and dense LDL, Kwon et al.^27^ described that this particle was independently associated with the incidence and extension of CAD in a Korean population, confirmed by subsequent studies.^28,29^ Studies have also reported a negative correlation between LDL size and risk of acute myocardial infarction.^30,31^ Similarly, small and dense LDL was associated with increased TG and decreased HDL-C levels.^32^ Therefore, results presented in this study showed for the first time that the LAP was significantly and robustly associated with the more atherogenic small LDL particle in Brazilians subjects above 30 years of age and moderate cardiovascular risk.

Contrary to high LDL-C level, low HDL-C level is accepted as an independent risk factor for CVD.^22,23,32^ Currently, it has been proposed that reverse cholesterol transport and other HDL properties such as antithrombotic action, endothelial function, and antioxidant and anti-inflammatory activities depend on HDL size.^33^ Larger HDL particles have a higher content of Apo AI and are described as more effective in reverse cholesterol transport.^3^ Asztalos et al.^32^ showed that a predominance of small, rather than large HDL particles, increased the risk of coronary heart disease. It was also suggested that small HDL particle size is associated with several features of the metabolic syndrome and risk of CAD.^34^ Our results showed a negative relationship of LAP with larger HDL and a positive relationship with smaller HDL particles. This profile is in agreement with the increased concentrations of HDL-C levels in subjects with lower LAP, although no correlation was found between LAP and Apo A1. Together with the LDL results, it reinforces the role of LAP as a surrogate marker for atherogenic lipoprotein subfractions.

In addition, our findings also showed a positive linear trend between NEFA values and LAP. Epidemiological studies have reported an association between NEFA and the risk of diabetes mellitus.^35,36^ Increased concentrations of NEFA in individuals with visceral obesity contribute to the development of various disorders such as peripheral insulin resistance, dyslipidemia, and b-cell apoptosis.^37^ Our data showed NEFA values similar to or higher than the values reported in the literature.^38,39^ This is compatible with the increased values also observed for glucose, insulin and HOMA-IR, independent of sex in our study. Linear trends between LAP and fasting glucose, insulin and HOMA-IR confirm that this index is associated with multiple glucose- and cardiovascular-related dysfunctions. Previously, Sambataro et al.^40^ showed that insulin sensitivity is not limited to dysfunction of fasting glucose and insulin and that lipid metabolism may affect this sensitivity. Therefore, the ability of LAP to simultaneously identify changes in glucose and lipid metabolism can expand the clinical relevance of this index.

This study had some limitations. The most significant one is that this study was conducted only in individuals with at least one cardiovascular risk factor, i.e., hypertension, diabetes mellitus or dyslipidemia. This suggests that the association found here might not be valid for health people. On the other hand, unfortunately, early diagnosis of dyslipidemia and changes in glucose metabolism are common events in young adults. Thus, more individuals would benefit from the inclusion of LAP in screening and monitoring of cardiovascular risk. Second limitation is the evaluation of previous cardiovascular events by clinical data and changes in the ECG. Although it is known that these data do not necessarily reflect the absence of coronary disease, in clinical practice, individuals are not submitted to complementary tests, such as provocation test to detect myocardial ischemia, if the initial evaluation indicates low cardiovascular risk. In screening protocols, ECG, in combination with complementary clinical and biochemical data, is the first instrument used because of its low cost. However, we admit that cardiovascular disease cannot be excluded in these individuals. And third, individuals included in this study were under statin (27.9%) and fibrate (2.6%). These drugs exert direct and indirect actions in lipid metabolism promoting changes in TG, a component of LAP. Despite that, these individuals were receiving the same drug treatment (in terms of type and posology) for at least 30 days prior to the study.

Methods for the measurement of emerging cardiovascular risk factors are generally complex and expensive, and hence could not be used in large-scale studies. LAP is a low-cost, easily measured variable that could be used to establish causal effects on clinical outcomes. So, the positive results from clinical trials and prospective cohort studies using this instrument are expected to encourage new approaches to estimate CVD risk.

## Conclusions

In conclusion, our results showed that the LAP index was associated with an atherogenic lipoprotein profile in Brazilian subjects, such as TC, HDL-C, Apo B, small HDL, small LDL and LDL size. It is plausible to suggest that the LAP may be a useful and simple clinical marker for assessment of cardiometabolic risk factors.
